# Network cartographs for interpretable visualizations

**DOI:** 10.1038/s43588-022-00199-z

**Published:** 2022-02-24

**Authors:** Christiane V. R. Hütter, Celine Sin, Felix Müller, Jörg Menche

**Affiliations:** 1 Department of Structural and Computational Biology, Max Perutz Labs, University of Vienna, Vienna, Austria; 2 CeMM Research Center for Molecular Medicine of the Austrian Academy of Sciences, Vienna, Austria; 3 Vienna BioCenter PhD Program, a Doctoral School of the University of Vienna and the Medical University of Vienna, Vienna, Austria; 4 Faculty of Mathematics, University of Vienna, Vienna, Austria

**Keywords:** Scientific data, Network topology, Regulatory networks

## Abstract

Networks offer an intuitive visual representation of complex systems. Important network characteristics can often be recognized by eye and, in turn, patterns that stand out visually often have a meaningful interpretation. In conventional network layout algorithms, however, the precise determinants of a node’s position within a layout are difficult to decipher and to control. Here we propose an approach for directly encoding arbitrary structural or functional network characteristics into node positions. We introduce a series of two- and three-dimensional layouts, benchmark their efficiency for model networks, and demonstrate their power for elucidating structure-to-function relationships in large-scale biological networks.

## Abstract (web summary)

Networks offer a powerful visual representation of complex systems. This study introduces network visualizations that are easy to interpret and can help explore large datasets, such as the map of all molecular interactions in the cell.

## Main

Networks are used to investigate a wide range of technological, social and biological systems^1^. Key factors for their success are the availability of powerful mathematical and computational analysis tools, but also their intuitive visual interpretation. For example, the central position of genes within molecular networks indicates essential cellular processes^2^, densely connected clusters represent functional complexes^3^, and global patterns, such as the ring-like architecture of co-regulation networks, have been found to reflect principles of cellular organization^4^. However, the full potential of network visualizations for exploring complex systems is limited by several conceptual and practical challenges. (1) Networks do not have a natural two- or three-dimensional (2D or 3D) embedding. Any network layout thus involves a choice of which aspects of the high-dimensional pairwise relationships are visually represented, and which are not. (2) In widely used layout algorithms, such as force-directed methods, this choice is made in an implicit and thus intransparent fashion, often based on subjective, esthetic criteria. This lack of a clear relationship between structural network characteristics and node positioning makes the resulting layouts difficult to interpret. (3) Likewise, there are no layout algorithms available that allow for explicitly representing a given network characteristic. (4) Finally, the big size of many real-world networks is a key limiting factor for producing comprehensible layouts, leading to proverbial hair-ball visualizations. In this Brief Communication we introduce a framework for generating network layouts that address these challenges by using dimensionality reduction to directly encode network properties into node positions. Not only can structural network properties be visually encoded in this fashion, but also external information reflecting the functional characteristics of nodes or links.

**Fig. 1:**
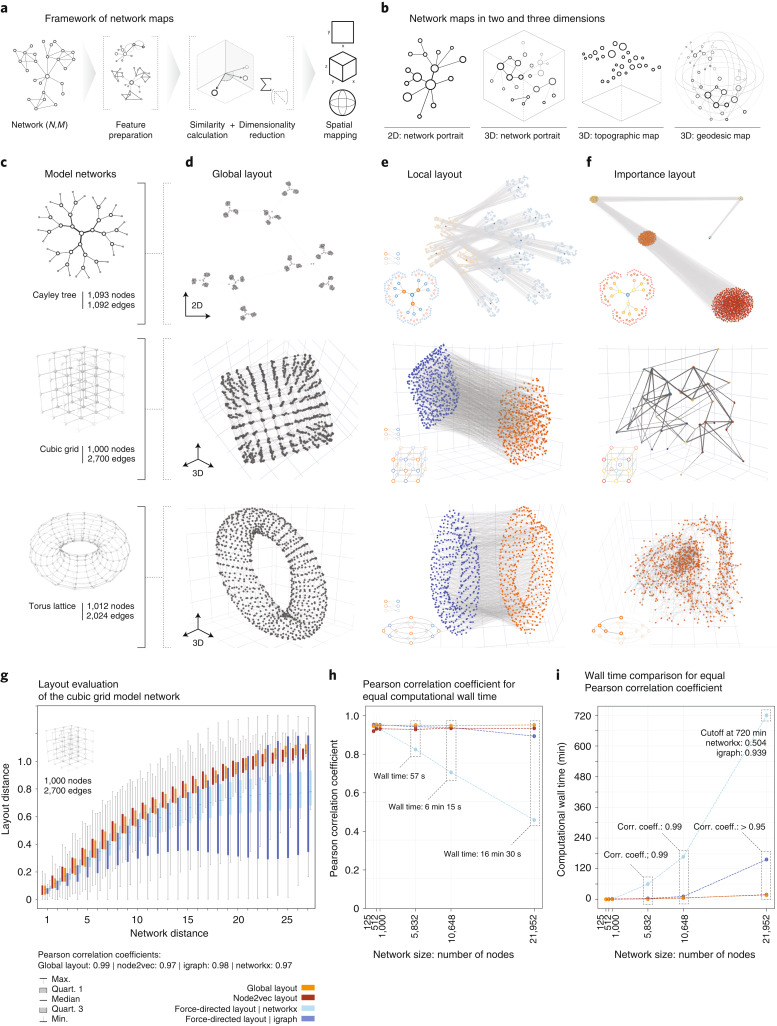
Framework of interpretable network maps. **a**, Overview. A node similarity matrix reflecting any network features to be visually represented is embedded into 2D or 3D geometries using dimensionality reduction methods. **b**, Schematic depiction of the resulting four types of network map: 2D and 3D network portraits directly use the outputs of the dimensionality reduction; topographic and geodesic maps incorporate an additional *z* or radial variable, respectively. **c**, The network models used for benchmarking: Cayley tree, cubic grid and torus lattice. **d**–**f**, Model network portraits based on global (**d**), local (**e**) and importance (**f**) layouts. The global layouts recapitulate the expected global shape according to pairwise node distances. The local layouts reveal bi- and multipartite network structures. The importance layouts cluster nodes with similar structural importance. **g**, Comparison of network-based and Euclidean layout distance for all node pairs in a cubic grid (*N* = 1,000) for the global layout, two force-directed algorithms and node2vec. All layouts achieve high correlation (Pearson’s *ρ*_glob_ = 0.99, *ρ*_node2vec_ = 0.97, *ρ*_force,nx_ = 0.97, *ρ*_force,igraph_ = 0.98). Boxes summarize values of all *n* node pairs at network distance *d*, with *n* ranging from *n* = 4 at distance *d* = 27 (for corner node pairs) to *n* = 46,852 for *d* = 9. Whiskers denote the values for the minimum, first, second and third quartiles and maximum. **h**, Comparison of the final correlations for cubic grids of increasing size when limiting the wall clock running time of the algorithms to the running time of the global layout. **i**, Computational wall times that the respective algorithms require to achieve the same correlation as the global layout for cube grids of increasing size. Source data

We propose the following procedure (Fig. 1a). For a given network, we first compile a set of *F* features for each of *N* nodes, incorporating any structural or functional characteristic we wish to be visually reflected in the final layout. The resulting (*N* × *F*) feature matrix is then converted into an (*N* × *N*) similarity matrix, which serves as input to dimensionality reduction methods to compute 2D or 3D embeddings. These embeddings can either be used directly as node coordinates, resulting in network layouts we termed portraits. Alternately, embeddings on 2D surfaces can be further extended towards 3D topographic or geodesic maps by using the third dimension for an additional variable of choice. The topographic map extends a flat 2D embedding by an additional *z* coordinate, and geodesic maps introduce an additional radial coordinate in spherical embeddings. In total, our framework thus offers four different maps in two and three dimensions (Fig. 1b). The key advantage of our framework, offering both versatility and interpretability, is its ability to incorporate and explicitly display various desired node characteristics or node pair relationships. We implemented five examples that demonstrate the diversity of potential layouts. (1) The global layout uses network propagation for an efficient, high-resolution representation of pairwise network distances. (2) The local layout emphasizes similar connection patterns between node pairs. (3) The importance layout combines several metrics for the overall importance of a node, such as degree, betweenness, closeness and eigenvector centrality. (4) Functional layouts depict node similarities according to external node features. (5) Combined layouts allow for tuning between layouts that are dominated by either structural or functional features.

To illustrate and benchmark our framework, we first applied it to easily interpretable model networks: (1) a Cayley tree, (2) a cubic grid and (3) a torus lattice (Fig. 1c). The Cayley tree is organized in hierarchical levels. All nodes except for those in the outermost level have the same number of neighbors (degree *k* = 3), and all nodes within the same level have identical centrality values. The cubic lattice contains four structurally different node groups: nodes at the corner (*k* = 3), along the 12 edges (*k* = 4), on the six faces (*k* = 5) or in the interior (*k* = 6). In the torus lattice, all nodes are equivalent in terms of all structural characteristics, including their degree (*k* = 4) and centrality metrics. Note that the definition of none of the model networks involves any spatial embedding, so, in principle, no layout is in any formal sense more correct than any other. However, for all three network models, canonical layouts in two and three dimensions, respectively, exist, offering an intuitive visualization of their global architecture. Our global layout provides a good approximation for these idealizations (Fig. 1d). The local and importance layouts produce entirely different results, each highlighting distinct structural aspects of the model networks. In the local layouts, the nodes are sorted into groups with shared neighbors (Fig. 1e). This layout reveals bi- and multipartite network structures, resulting in two clusters in the lattice-based networks (cube and torus), and in alternating patterns reflecting the ternary structure of the Cayley tree. The importance layout identifies groups of nodes with the same network centralities (Fig. 1f). In the Cayley tree, all nodes of the same hierarchy are clustered, and in the cubic grid, nodes of the same type (corner, edge, face nodes) and layer are grouped. In the torus, all nodes have equivalent structural roles, thus resulting in a uniform point cloud.

**Extended Data Fig. 1:**
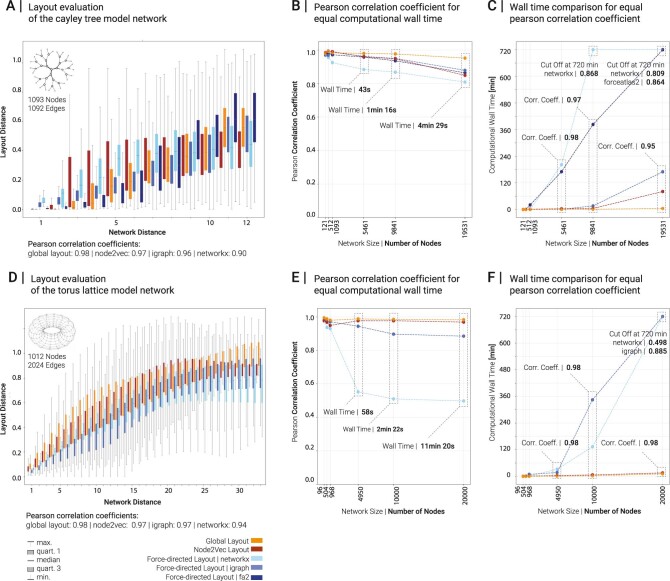
Benchmarking different layout algorithms for model networks. **A** Comparison of pairwise node distances in the layout and pairwise network distance for a Cayley tree with *N* = 1093 nodes and *M* = 1092 links. Boxes summarize values of all *n* node pairs at network distance *d*, with *n* ranging from *n* = 1092 at distance *d* = 1 to *n* = 177,147 for *d* = 12. Whiskers denote the values for the minimum, first, second, third quartiles and maximum. **B** Comparison of the final Pearson correlation coefficient between network and layout distance that the different algorithms achieve in the same computational wall time as the global layout for Caley trees with sizes ranging from 121 to 21,952 nodes. **C** Comparison of the computational wall times that the different algorithms require to reach the same correlation coefficient as the global layout. For network sizes of 10,000 nodes and above, the force-directed algorithms do not reach the target correlation within the maximum simulation time of 12 h. **D, E, F** Same as A,B,C for torus lattice model networks (*N* = 1012 nodes; *M* = 2024 links). Boxes in D summarize values of all *n* node pairs at network distance *d*, with *n* ranging from *n* = 484 at distance *d* = 33 to *n* = 21,296 for *d* = 12. Whiskers denote the values for the minimum, first, second, third quartiles and maximum. Source data

The global layout incorporates random walk-based features similar to the graph embedding method node2vec^5^. Also, for small to moderate network sizes, standard force-directed algorithms^6^ produce layouts that recapitulate network distances between node pairs. We can therefore use these algorithms as performance benchmarks. Figure 1g shows good overall correlations between network-based node distances in cubic lattice networks and the respective layout distances (Extended Data Fig. 1). A comparison of the correlations obtained for the same computational running time shows a substantial drop for force-directed algorithms as the network size increases (Fig. 1h). Conversely, force-directed methods are orders of magnitudes slower for fixed layout quality (Fig. 1i).

**Fig. 2:**
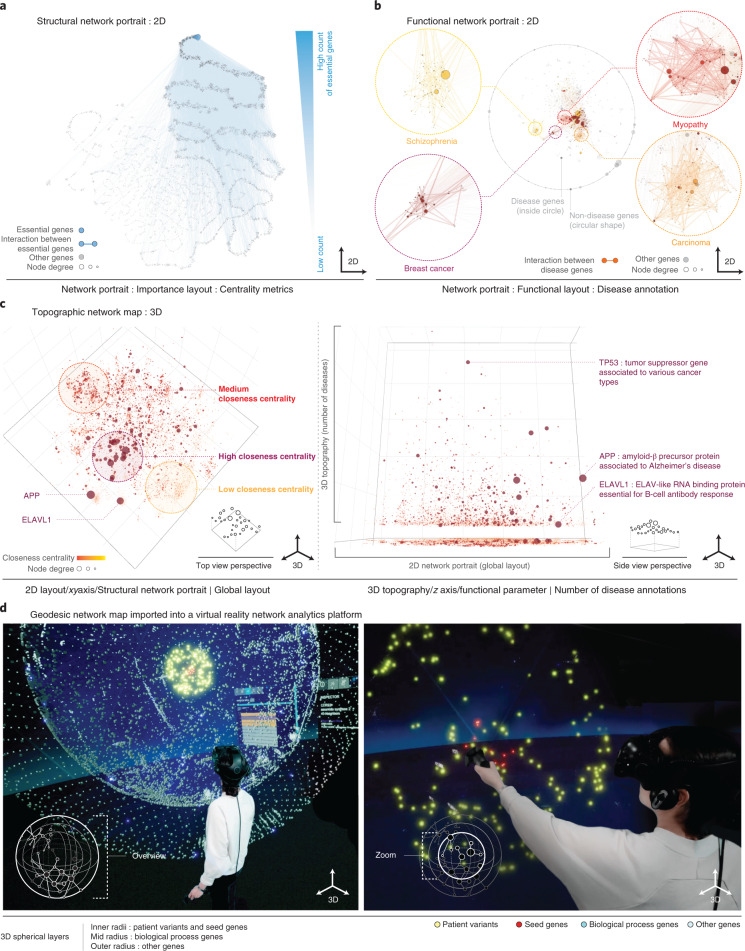
Application to a large-scale, real-world biological network. **a**, Structural network portrait of the human interactome based on the importance layout. Essential genes and links between them are shown in blue and aggregate in the area of high centrality nodes (top right). **b**, Functional network portrait based on disease association similarity. Four diseases are highlighted. Only links between disease genes are shown. Although most disease genes are located in four clusters (links shown by thicker lines), a smaller number of pleiotropic genes associated with multiple diseases is located at the center of the network (Extended Data Fig. 4b). **c**, Topographic network map in top view (left) and side view (right) obtained from a 3D interactive visualization. The *x–y* plane is based on a 2D global layout, and the *z* axis displays the number of diseases associated with a particular gene. **d**, Green-screen composition of a user exploring a geodesic network map in a virtual reality environment^13^. Nodes are distributed on different spherical layers that reflect different biological roles. The center contains nodes to be functionally annotated, the enclosing layers contain genes associated with similar diseases and involved in relevant biological processes, respectively. Each individual layer is based on a functional layout emphasizing biological similarity, allowing the user to quickly identify the biological context of individual genes and their interactome neighborhood. Source data

**Extended Data Fig. 2:**
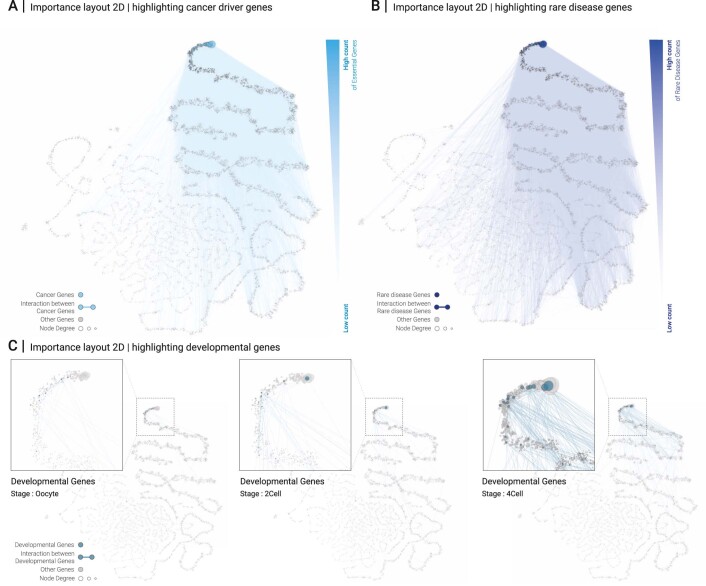
Importance layout of the interactome with different functional gene annotations highlighted. **A** Cancer driver genes and links between them are shown in blue, revealing a clear agglomeration at the top right, corresponding to high centrality nodes. **B** Same as A, highlighting rare disease genes. **C** The three visualizations highlight genes expressed in the three earliest developmental gene stages, from a single oocyte, to 2-cell and to 4-cell stages, respectively (left to right). The visualizations suggest that early stage development starts out at the most highly central genes, before involving more and more peripheral genes. This trend has, to the best of our knowledge, not been documented before and warrants further, rigorous evaluation and validation. Source data

**Extended Data Fig. 3:**
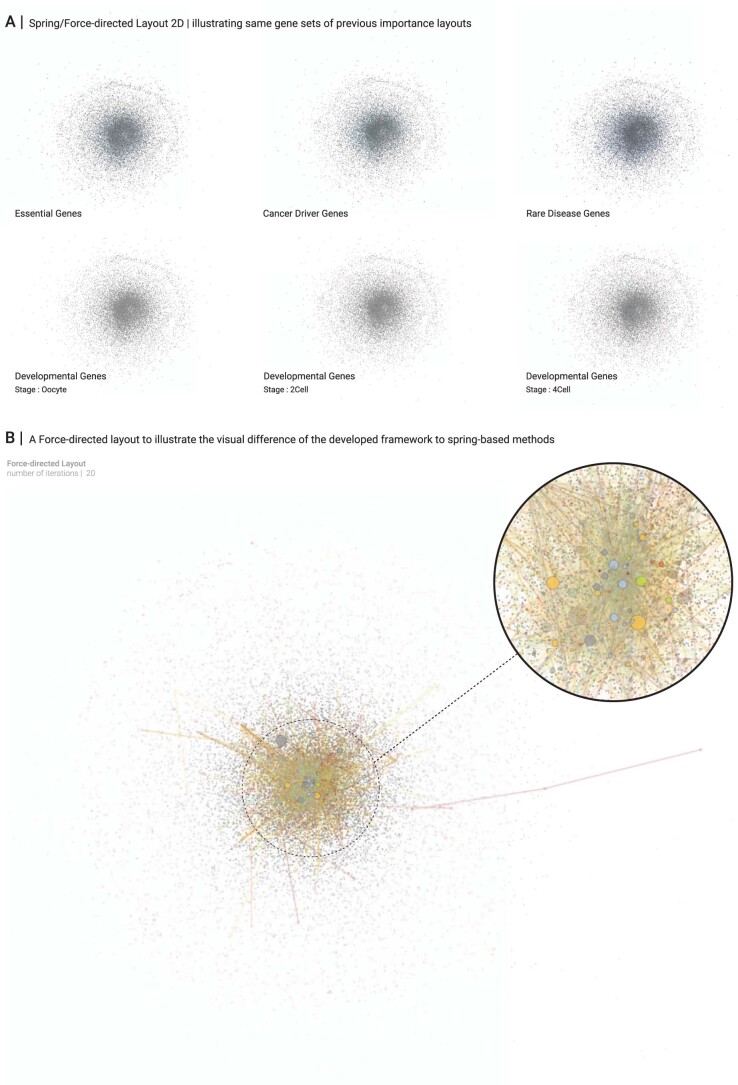
Force-directed layout of the interactome. **A** Layouts with different functional gene sets highlighted. Colored nodes show the same gene sets as in the importance layouts in Fig. 2A and Extended Data Fig. 2. The correspondence between network centrality and biological importance cannot be extracted from these visualizations. **B** Layout with genes associated with neurofibromatosis and related diseases being colored as in Extended Data Fig. 6. The force-directed layout does not allow for visually discerning either connections within the respective diseases, nor between them.

**Extended Data Fig. 4:**
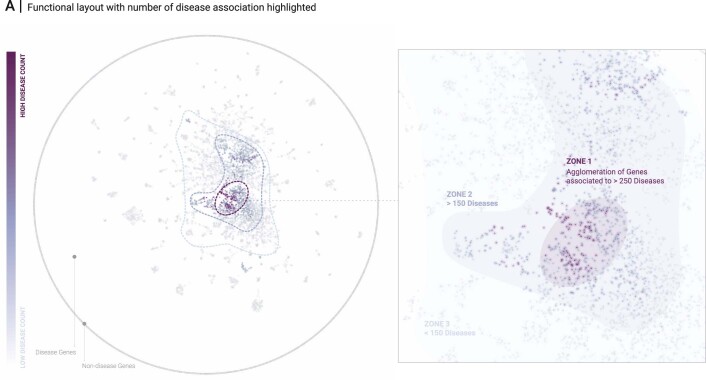
Functional network portrait for exploring genes with multiple disease associations. Functional network layout highlighting the number of diseases that genes are associated with using a gradient, from light (low disease count) to dark colors (that is high disease count). In combination with Fig. 2a, the visualization confirms that pleiotropic genes, that is genes associated with a high number of diseases, tend to be located in a separate area in the center of the functional layout. Source data

**Extended Data Fig. 5:**
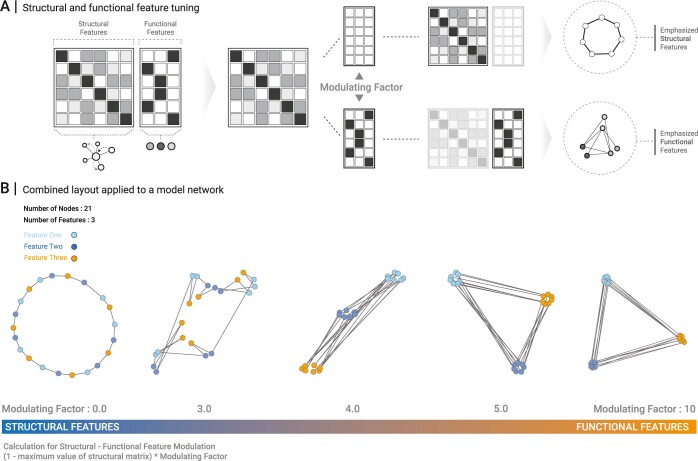
Combined structural and functional layout. **A** Illustration of the method for generating layouts that combine structural and functional features in a tunable fashion. The structural aspect of the layout is derived from the global layout, where each node in the network is represented by a feature vector containing random walk visiting frequencies to all other nodes. The functional aspect is then introduced by adding an additional column for each functional feature to be included in the layout, for example associations with different diseases. These functional columns contain values ‘1’ or ‘0’, depending on whether a particular node is associated with the respective feature (value ‘1’) or not (value ‘0’). Scaling the functional columns by a factor *m* ≥ 0 allows to modulate between purely structural layouts (*m* = 0) and layouts that are increasingly dominated by the functional annotations (*m* > 0). **B** Application of the method to a simple model network with ring structure three node annotations, indicated by different colors. As the modulation factor increases from *m* = 0 to *m* = 10, the layout transitions from a purely structural one, to one dominated by the node annotations alone.

**Extended Data Fig. 6:**
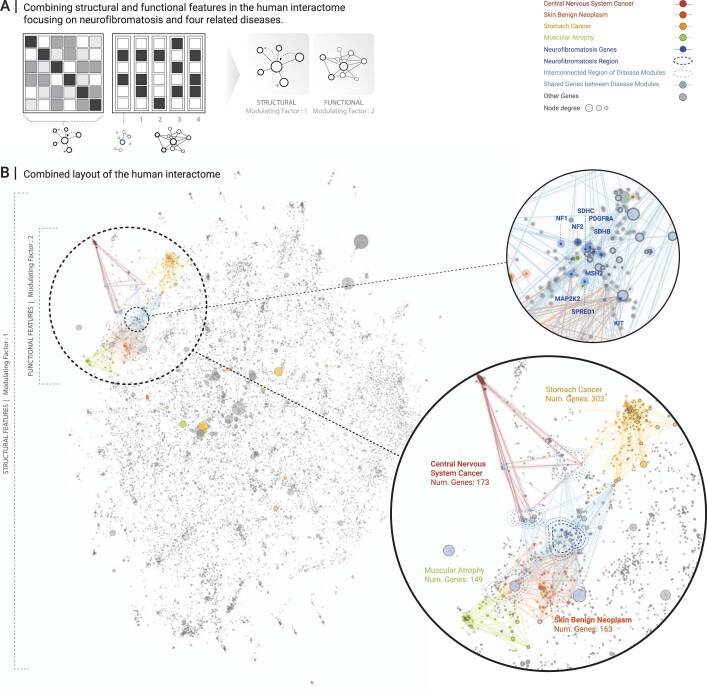
Combining structural and functional features of the interactome in the context of neurofibromatosis. **A** Illustration of the method for combining structural and functional features. First, a feature vector as in the global layout is constructed for each node, representing the structural aspect of the layout. The functional aspect is introduced by five additional columns with values ‘1’ or ‘0’ indicating whether a particular gene is associated with any of the five diseases of interest (value ‘1’) or not (value ‘0’). The functional columns are then scaled using a modulation factor *m*, such that *m* = 0 recapitulates the purely structural global layout, and increasing values of *m* lead to increasingly localized clusters of genes associated with the same diseases. **B** Combined structural and functional layout (*m* = 2) of the human interactome highlighting genes associated with neurofibromatosis and four related diseases. Neurofibromatosis (12 genes, shown in dark blue) is positioned in the center. Genes that are shared between disease modules, as well as links connecting genes of different modules are shown in light blue. The layout can be used to examine potential molecular mechanisms that underlie relationships observed between diseases of interest. Here, the relationship is based on shared clinical manifestations, whose molecular underpinnings remain largely unknown in the case of neurofibromatosis. Source data

We next apply our framework to a large real-world network. The human interactome consists of *N* = 16,376 nodes and *M* = 309,355 links, representing proteins and their physical interactions that underlie biological processes^7,8^. Although several structure-to-function relationships in the interactome are well documented^9^, they are difficult to decipher visually from conventional layouts. Our framework offers a solution to this challenge. Figure 2a shows a 2D network portrait of the interactome in the importance layout. Visual inspection of 2,918 known essential genes reveals a relationship between their structural importance within the interactome and their biological importance. Cancer driver genes, rare disease genes and genes involved in early development show the same trend (Extended Data Fig. 2a–c). Although this finding represents one of the cornerstones of network biology^2^, it could not be derived from standard layouts (Extended Data Fig. 3a). Similarly, the agglomeration of genes associated with the same disease in local interactome neighborhoods is well documented^10^, yet remains hidden in standard layouts (Extended Data Fig. 3b). We can use functional network portraits to visualize disease-associated genes and their interconnectivity (Fig. 2b). Although the node placement is purely driven by a functional characteristic, the underlying network structure can be inspected through the links. This supports the identification of structure-to-function relationships in an iterative cycle of data visualization, hypothesis generation and validation. In addition to disease gene interconnectivity, Fig. 2b also shows a prominent cluster of highly connected genes associated with multiple diseases (Extended Data Fig. 4). Finally, we can also generate layouts in which the node positions are determined by a combination of structural and functional features (see Extended Data Figs. 5 and 6 for applications to a model network and the interactome).

Network maps with an additional quantity of interest depicted in the third dimension can be used to build application-specific visualizations. Figure 2c shows a 3D topographic map of the interactome, with a global layout on the *x–y* plane and the number of disease associations on the *z* axis, highlighting, for example, the prominent role of the tumor suppressor TP53 in many cancers^11^. The top view reveals several localized node clusters, which correspond to provincial hubs and their respective neighbors^12^. The side view shows the prominent role of the provincial hubs for diseases and their relationships, such as amyloid precursor protein (APP) and ELAV-like RNA binding protein (ELAVL1), which are located at the center of the respective interactome neighborhoods that are perturbed in the associated diseases^13^.

Figure 2d demonstrates how our framework can be utilized for generating network maps customized to the interactive annotation of rare genomic variants in a virtual reality environment^14^. The center sphere of the geodesic map contains 13 candidate genes that are suspected to cause a rare genetic disease in a particular patient. The enclosing spheres represent genes implicated in similar phenotypes or involved in related biological pathways, respectively, in a functional layout reflecting biological similarity. This allows for an efficient manual inspection of the biological context of the candidate genes.

The flexibility of our framework enables the development of customized network visualizations for a broad range of applications. In biology, for example, the introduced layouts may enhance existing tools for the integration and interpretation of diverse omics datasets^15–19^. Note that visual inspection alone will rarely suffice to conclusively show the presence of an observed structure-to-function relationship in a given network. Any hypothesis derived from a particular visualization thus requires an additional, more rigorous evaluation outside of our framework, for example, by statistical or experimental means.

## Methods

### A framework for creating interpretable network layouts and maps

Our pipeline consists of four basic steps. (1) The network of *N* nodes and *M* links is supplied in the form of a link list. (2) For each node in the network, we construct a vector of *F* features, resulting in an (*N* × *F*) feature matrix. The particular features that are used determine the layout. We introduce five such layouts, termed ‘global’, ‘local’, ‘importance’, ‘functional’ and ‘combined’ layouts, as detailed in the next sections. (3) The feature matrix is converted into an (*N* × *N*) similarity matrix, which serves as input for dimensionality reduction algorithms. The utility of dimensionality reduction techniques for network embedding is increasingly recognized, in particular for classification tasks and more recently also for visualizations^20^. We implemented the popular tools *t*-distributed neighbor embedding (*t*-SNE)^21^ and uniform manifold approximation and projection (UMAP)^22^, which offer embeddings in 2D and 3D Euclidean space, as well as embeddings on 2D surfaces, such as a sphere. (4) The node coordinates can either be used directly to lay out the network or can be further enhanced by an additional third dimension in the case of 2D embeddings. We termed the direct layouts ‘portraits’. Flat embeddings in 2D Euclidean space can be expanded into 3D topographic maps by using an additional, freely selectable variable as the *z* coordinate. Similarly, we can enhance embeddings on the surface of a sphere by introducing an additional radial variable, resulting in geodesic maps.

### Global layout

In the global layout, each node is equipped with *N* features representing its network-based distances to all nodes in the network based on a random walk with the restart propagation method^23^. These random walk-based distances indicate how frequently a walker starting from node *i* and traveling along randomly chosen links will visit a given node *j*. Formally, we first determine the vector **p**_*i*_ containing the visiting frequencies *p*_*i,j*_ for all nodes *j* *∈* [1, *N*] starting from node *i* as seed for a random walk with restart probability *r*. These frequencies can be efficiently computed by matrix inversion according to the steady-state expression for a random walk with restart^24^. For all node pairs {*n*, *m*}, we then compute the cosine similarity *S*(*n*, *m*) between their respective visiting frequency vectors **p**_*n*_ and **p**_*m*_ and collect the results into an (*N* × *N*) similarity matrix *S*_glob_ that serves as input to the dimensionality reduction step of the pipeline.

### Local layout

The local layout is based on the similarity of nodes in terms of shared neighbors. Two nodes that are connected to the exact same set of nodes are considered maximally similar, whereas nodes that do not have any common neighbors do not have any similarity. We can determine this similarity directly from the adjacency matrix *A* of the network, defined as *A*_*i*,*j*_ = 1 if nodes *i* and *j* are connected, and *A*_*i*,*j*_ = 0 otherwise. For all node pairs {*n*, *m*}, we compute the cosine similarity between their corresponding columns *A*_*i*,*n*_ and *A*_*i*,*m*_, resulting in an (*N* × *N*) similarity matrix *S*_loc_ which serves as input to the dimensionality reduction step.

### Importance layout

The importance layout reflects the similarity of nodes in terms of their network centralities^1^. Network centralities measure the importance of a particular node according to its position within the network. Numerous centrality measures have been proposed, and we incorporated four of the most widely used into a feature vector. For each node *i* we compute its (1) degree (the number of neighbors), (2) closeness (its average network distance to all other nodes), (3) betweenness (how often it acts as a bridge along the shortest path between two other nodes) and (4) eigenvector centrality (measuring its dynamic influence), resulting in a 4D vector **c**_*i*_. For all node pairs {*n*, *m*}, we then compute the cosine similarity between their corresponding vectors **c**_*n*_ and **c**_*m*_, resulting in an (*N* × *N*) similarity matrix *S*_cent_, which serves as input to the dimensionality reduction step.

### Functional layouts

Functional layouts can be used to display node similarities in terms of external features, such as the disease annotations of genes in Fig. 2b. For a given feature matrix *F* with *F*_*i*,*j*_ = 1 if node *i* is annotated to feature *j*, and *F*_*i*,*j*_ = 0 otherwise, we compute the cosine similarity between all node pairs {*n*, *m*} using the respective rows *F*_*n*,*j*_ and *F*_*m*,*j*_, resulting in an (*N* × *N*) similarity matrix *S*_func_, which serves as input to the dimensionality reduction step.

### Combined layouts

Combined layouts allow for extrapolating between purely structural and functional layouts. We first construct a matrix with elements *p*_*i*,*j*_ as in the global layout above, representing the structural aspect of the final layout. For each functional feature that we wish to include, for example annotations to different diseases, we then add an additional column containing the values *F*_*i*,*j*_ = 1 if node *i* is annotated to feature *j*, and *F*_*i*,*j*_ = 0 otherwise. These functional columns can now be scaled by a factor *m* ≥ 0, thereby modulating between purely structural layouts (*m* = 0) and layouts that are increasingly dominated by the functional annotations (*m* > 0). Finally, for all node pairs {*n*, *m*}, we compute the cosine similarity *S*(*n*, *m*) between their vectors **p**_*n*_ and **p**_*m*_ and collect the results into an (*N* × *N*) similarity matrix *S*_comb_, which serves as input to the dimensionality reduction step of the pipeline.

### Implementation

We used the Python package networkx^25^ to generate the model networks and compute the network properties required in the different layouts, such as adjacency matrices and node centralities. The force-directed layouts were generated using the Fruchterman–Reingold algorithm^6^ as implemented in NetworkX and igraph^26^, respectively, and using ForceAtlas2^27^. Dimensionality reduction methods were implemented using the *t*-SNE^24^ and UMAP Python packages^25^, and the node2vec algorithm was implemented using the StellarGraph library^28^. Note that the implemented dimensionality reduction methods are not strictly deterministic, so that repeated calls may lead to slightly different outputs. To maximize the reproducibility, we therefore set a fixed random seed in the provided Python code.

To evaluate how well a particular layout algorithm reproduces network-based distances between nodes, we computed for all node pairs {*n*, *m*} the length of the respective shortest paths ${d}_{n,m}^{\rm{SP}}$ and their Euclidean distance ${d}_{n,m}^{\rm{Euc}}$ within the layout. The agreement between the two was then quantified using the Pearson correlation coefficient:

$${{r}} = {\frac{{\mathop {\sum}\limits_{\{ n,m\} } {({d}_{n,m}^{\rm{SP}} - {\mu }^{\rm{SP}})({d}_{n,m}^{\rm{Euc}} - {\mu }^{\rm{Euc}})} }}{{\sqrt {\mathop {\sum}\limits_{\{ n,m\} } {({d}_{n,m}^{\rm{SP}} - {\mu }^{\rm{SP}})^2} \mathop {\sum}\limits_{\{ n,m\} } {({d}_{n,m}^{\rm{Euc}} - {\mu }^{\rm{Euc}})^{2}} } }}}$$

where *µ*^SP^ and *µ*^Euc^ denote the respective mean values of network-based and Euclidean distances across all node pairs. We used the implementation contained in the numpy Python package^29^. Computational wall time was measured on computer hardware with a 2-GHz Quad-Core Intel Core i5 processor and 16 GB of RAM.

### Source data

Source Data Fig. 1 Statistical data.

Source Data Fig. 2 Layout data tables.

Source Data Extended Fig. 1 Statistical data.

Source Data Extended Fig. 2 Layout data tables.

Source Data Extended Fig. 4 Layout data table.

Source Data Extended Fig. 6 Layout data table.

## Figures and Tables

**Extended Data Fig. 7:**
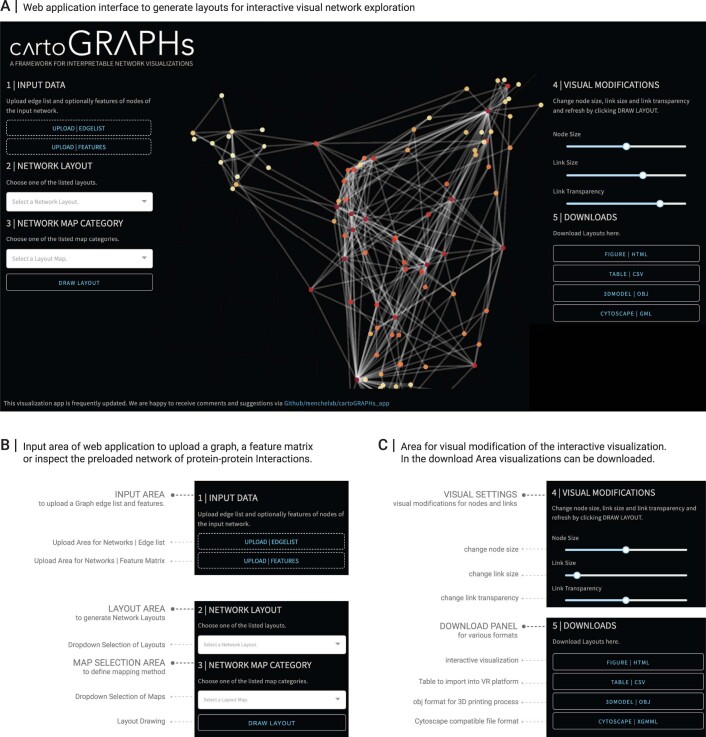
A Web application interface of the CartoGRAPHs framework. **A** Screenshot of the web application. **B** Input area for uploading network and functional node annotation data, selecting layouts and mapping types. **C** Areas for adapting the visualization and for downloading the final layouts in different formats, including interactive html files, XGMML files for further processing in the cytoscape software, and files for import into 3D softwares or a virtual reality (VR) analytics platform.

## Data Availability

All input files, together with the complete source code, have been deposited in a Zenodo repository^30^. The human interactome network was extracted from the HIPPIE database^31^, filtering for protein–protein interactions with at least one supporting PubMed article. Disease gene associations were taken from the DisGeNET database^32^ and mapped to disease categories according to Disease Ontology (DO)^33^. Functional gene annotations were derived from the ‘biological processes’ branch of the Gene Ontology (GO) database^34^. Essential genes were obtained from the Online Gene Essentiality (OGEE) database^35^, rare disease genes from OrphaNet^36^ and genes involved in early development from the EmExplorer database^37^. Source data are provided with this paper.

## Code availability

Python source code and input data for reproducing the results in this paper are publicly available from the Zenodo repository^30^. We also provide the code as a Python package on GitHub at https://github.com/menchelab/CartoGRAPHs, together with Jupyter notebooks including a quickstarter, as well as separate notebooks for reproducing each figure. The CartoGRAPHs framework can also be used as an interactive web application at www.cartographs.xyz and source code is provided at https://github.com/menchelab/cartoGRAPHs_app (Extended Data Fig. 7). As output, 2D and 3D network interactive images can be generated and downloaded in html format. Layouts can also be exported as XGMML files that can be loaded for further processing in the cytoscape software^38^ . Finally, we offer export in Wavefront OBJ format to be implemented into 3D printing processes or for exploring network maps in VRNetzer, a virtual reality platform^12^ for network visualization and analysis.
