# Supplementary material for: Network cartographs for interpretable visualizations
Source: Nat Comput Sci. 2022 Feb 24;2(2):84–9. doi: 10.1038/s43588-022-00199-z (PMC10766564; doi:10.1038/s43588-022-00199-z)
Supplement: Supplementary file 1 — Statistical data. [file 43588_2022_199_MOESM1_ESM.zip › Figure1/README_fig1_EDfig1.pdf]

READ ME

FOR FIGURE SOURCE DATA : FIGURE 1 / EXTENDED FIGURE 1

ADDITIONAL INFORMATION

The files provided in folder Figure1.zip and EDFigure1.zip represent values calculated during the benchmarking process. We provide calculated values for different steps of the calculation. In the first step we calculate layout distances for each layout algorithm to be compared to initial network distances in figure 1G, Extended Data Figure 1A and 1D. In the second step we calculate Pearson correlation coefficients and computational wall time for comparison in Figure 1H,I and Extended Data Figure 1B,C and 1E,F.

The files “net_sizes_*.txt” contain a list of network model sizes, representing the numbers of nodes of each network model.

Files starting with “dist” contain calculated distances between node pairs for each network model. The name of a file includes the layout algorithm, the number of nodes and the name of the network model, such as for example “dist_spring_nx_1000_cube.txt”. Hereby “dist” refers to the file content of “layout distances”, “spring_nx” refers to the layout algorithm used and “1000_cube” represents the number of nodes and architecture of the network model. The files entitled “dist_network_*” contain network distances (i.e. shortest paths for node pairs) of the respective network model and size.

Filenames starting with “d_netsize” contain values or correlation factors and computational wall time in seconds, respectively. The files are named according to the dictionary structure of content, for example “d_netsize_time_nx_cube.txt” contains computational wall time values for each network size of the layout algorithm “nx” in form of a dictionary, where keys represent network size and values represent computational wall time. The same principle applies for correlation coefficients, where “time” is replaced by “corr”.

INSTRUCTIONS

The file contents represent calculation steps in the process of benchmarking three network models.
An exemplary section, where to use the precalculated values from the supplied text files, is contained in the notebook “cartoGRAPHs_ManuscriptFigure_1G_Extended1AD.ipynb”.

ABBREVIATIONS

net ... Network model
sizes ... Number of Nodes of a Network
corr ... Pearson Correlation Coefficient
global ... Global layout
nx ... network
ig ... iGraph
fa ... Force Atlas2
nodevec ... Node2vec
