# Supplementary material for: Network cartographs for interpretable visualizations
Source: Nat Comput Sci. 2022 Feb 24;2(2):84–9. doi: 10.1038/s43588-022-00199-z (PMC10766564; doi:10.1038/s43588-022-00199-z)
Supplement: Supplementary file 2 — Layout data tables. [file 43588_2022_199_MOESM2_ESM.zip › Figure2/README_LayoutTables.pdf]

READ ME

LAYOUT TABLES

The provided layout tables contain node positions and colors for each network node.
The columns are structured according to file requirements of the Virtual Reality network analytics platform VRNetzer (Pirch et al., Nature Communications, 2021).

Each row represents one node of a network. Columns contain node ID, coordinates for x-, y- and z-values, color values (r,g,b,a for red,green,blue,alpha) and a layout name. Coordinates should be of type float, RGBA values should be provided as integers, node ID and layout names as strings.

An exemplary line of a layout table:
(first line = description; second line = actual content)

NodeID	x-pos	y-pos	z-pos	R	G	B	A	Namespace
**8473**	**0.4993**	**0.4544**	**0.640**	**188**	**20**	**26**	**100**	**3dportrait**

The layout table files can be used with an import function that is provided within the python module. The node ID is required for identification for obtaining links from the additional edge list. The alpha values and name columns are specific to the Virtual Reality platform file format.
